# Protocol of safe vaccination against COVID‐19 in patients with high risk of allergic reactions

**DOI:** 10.1002/clt2.12152

**Published:** 2022-05-17

**Authors:** Jan Romantowski, Jerzy Kruszewski, Oskar Solarski, Andrzej Bant, Andrzej Chciałowski, Ilona Pietrzyk, Patrycja Sańpruch, Aleksandra Górska, Marta Chełmińska, Agata Knurowska, Marika Gawinowska, Ewa Jassem, Marek Niedoszytko

**Affiliations:** ^1^ Department of Allergology Medical University of Gdansk Gdańsk Poland; ^2^ Department of Infectious Diseases and Allergology Military Institute of Medicine Warsaw Poland; ^3^ Department of Allergology Chmielnik Hospital Chmielnik Poland; ^4^ II Department of Radiology Medical University of Gdansk Gdańsk Poland; ^5^ Department of Pneumonology Medical University of Gdańsk Gdańsk Poland

**Keywords:** allergen, coronavirus, immunity, sars‐cov‐2, vaccination

## Abstract

**Background:**

Sars‐CoV‐2 infections are hazardous, especially to the elderly and patients with comorbidities. With no efficient treatment available, newly developed vaccines are the only way to change the course of the pandemic. However, reports of allergic reactions resulted in some patients and practicing physicians being concerned about the safety of vaccine administration, particularly in people with severe anaphylactic reactions to multiple or unknown factors in their medical history.

This study aimed to develop an allergic work‐up protocol based on skin prick tests (SPT), intradermal testing (IDT) and intramuscular provocations, and desensitisation which may contribute to diagnosis and management of anti‐COVID‐19 vaccine allergy.

**Methods:**

Two hundred and eighty‐five patients were enrolled. Two hundred and five of them entered the study based on severe anaphylactic reaction to unknown or multiple factors in their medical history which disqualified them for standard treatment. Another 80 patients were enrolled after developing an allergic reaction to the first dose of one such vaccine. In all subjects, SPT and IDT were performed. Serum tryptase was assessed in 79 patients randomly chosen from the study group.

**Results:**

Two hundred and seventy‐seven patients with negative tests were given a vaccine without complications. Seven patients had positive skin tests. In two cases, tests confirmed Comirnaty allergy, while the other five confirmed solely skin sensitisation with no exposure prior to the study. Six patients with positive tests received titrated challenge using desensitisation protocol with a reasonable tolerance. One patient did not consent to desensitisation and one patient resigned despite negative tests. Overall, 283 (99%) patients were vaccinated using this newly developed protocol. Patients with adverse reactions to the first dose of the vaccine before the study had a significantly lower basal serum tryptase concentration (*p* = 0.001).

**Conclusion:**

Skin tests with anti‐COVID‐19 vaccines are a useful tool in the vaccination protocol. This protocol enables safe immunisation of high‐allergy‐risk patients even in cases of positive skin tests.

## 1 BACKGROUND

Since December 2019, the pandemic caused by the novel coronavirus disease 2019 (COVID‐19) has been the most pressing global health crisis. With no effective therapy for Sars‐CoV‐2 infection, vaccination is currently the most dependable strategy to end the pandemic by acquiring ‘herd immunity’. Several SARS‐CoV‐2 vaccines have been approved around the globe, including mRNA (i.e., Comirnaty® and Spikevax®), and viral vector (COVID‐19 Janssen and Vaxzevria®) vaccines.^1^ All of them have demonstrated high safety and efficacy in preventing severe COVID‐19 infection in clinical trials.^2^, ^3^, ^4^, ^5^ Nevertheless, in the first 48 h of the vaccination programme in the United Kingdom, two reports of severe allergic reactions that required epinephrine treatment were published.^6^ Shortly after that, the Centers for Disease Control and Prevention (CDC) in the United States advised that all patients should be observed for 15 min after Sars‐CoV‐2 vaccination and that vaccination staff must be trained to manage anaphylaxis.^7^ The CDC provided further recommendations ‘that persons who have had an immediate allergic reaction of any severity to any vaccine or injectable therapy (intramuscular, intravenous, or subcutaneous) should discuss the risk of receiving the vaccine with their doctors and be monitored for 30 min afterwards’. In addition, patients who have an immediate (within 4 h) or severe allergic reaction to an mRNA Sars‐CoV‐2 vaccine should not receive a second dose.^8^ Similar precautions were taken with every authorised vaccine. This approach was met with reluctance in societies, especially after a few descriptions of severe post‐vaccination allergic and adverse reactions.^9^, ^10^, ^11^

Given the importance of the vaccination programmes in fighting the pandemic, understanding the allergic reactions and developing proper protocols for all allergic patients is crucial to balance a high vaccination rate with safety.

The most widely used approach to allergy work‐up protocols was to perform polyethylene glycol (PEG) or polysorbate skin testing, which are considered to be the most allergenic exipients of anti‐COVID‐19 vaccines.^12^, ^13^ Skin prick tests (SPT), intradermal tests (IDT) and to some extend basophil‐oriented in vitro tests were found reliable in detecting PEG and polysorbate allergy.^14^, ^15^, ^16^ However, less data is available on IDT, and some suggest false positive results.^17^ Both SPT and IDT may also become negative over time, especially when the patient is not exposed to the particular excipient frequently.^14^, ^16^ Also, some studies suggest that even PEG‐allergenic patients can safely receive anti‐COVID‐19 vaccines containing PEG.^15^ SPT with undiluted vaccine is recommended, especially in patients who experienced anaphylaxis on the first dose. Nilsson et al. have already developed skin test, provocation and desensitization protocols for other vaccines.^18^, ^19^ Finally, in vaccination units, the anti‐COVID‐19 vaccine is easily obtainable and each vial usually contains some spare amount.

This study aimed to develop vaccine allergy work‐up protocols including management of their administration in patients with a high risk of allergy and those who experienced allergic reactions after the first of two vaccine doses.

## 2 METHODS

Patients were randomly enrolled upon informed consent according to the Polish Society of Allergology vaccination guidelines in three centres in Poland: Gdansk, Chmielnik and Warsaw.^20^ They consisted of two groups: (1) people with a history of anaphylaxis and drug allergy to at least two groups of drugs or to any vaccine or unknown allergen (guidelines suggest that these patients may be vaccinated with close monitoring, epinephrine at hand and venule cannulation); (2) those who experienced an anaphylactic reaction to the first dose of anti‐COVID‐19 vaccine and were disqualified from the second dose (vaccination was generally not recommended in this group). Each patient received an initial consultation with an allergologist. Anaphylaxis was assessed according to World Health Organization criteria.^21^ Each patient must have experienced one of the following: hypotension, bronchospasm, laryngeal involvement after exposure to probable allergen during 2 h post vaccination.

**FIGURE 1:**
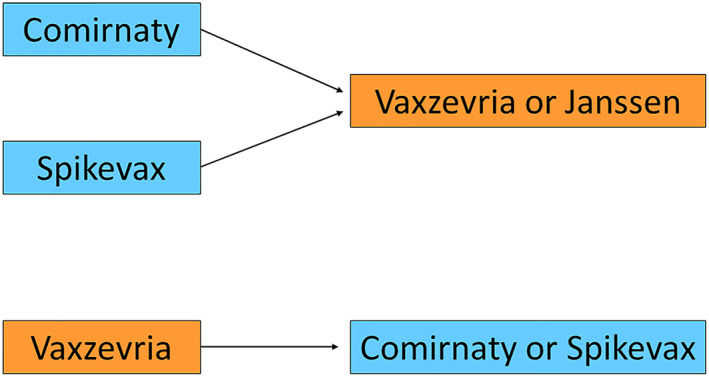
Applied schedule of changing the vaccine in the case of anaphylaxis after the first dose. COVID‐19 Janssen is a single‐dose vaccine so no decision was to be made in the case of an allergic reaction. In addition, all decisions were patient‐shared

In the first group,^1^ the possibility of PEG and polysorbate 80 allergies was assessed based on allergy history and a vaccine was chosen to possibly avoid the suspected allergen. In the second group,^2^ the vaccine was changed according to the schedule shown in Figure 1. The shared decision based on a patient‐physician discussion of all contributing factors was made in every case.

All patients were evaluated medically, including severe COVID‐19 risk factors (at least one of the following: age>59, hypertension, coronary heart disease, diabetes, obesity, active neoplastic disease).^22^ Additionally, in a randomly chosen subgroup of 79 participants, the basal serum tryptase concentration was analysed.

SPT and IDT were performed with the chosen vaccine in every patient according to Nilsson et al.^18^ For the SPT, the undiluted vaccine was used, and the positive criterion was set at 3 mm. The concentrations for IDT were 1:100 and 1:10 consecutively (volume 0.02 ml), and the positive criterion was an increase of the primary wheal by 3 mm. All tests were read after 15 min.

In the case of negative tests, the previously chosen vaccine was administered in the following manner: placebo, 10% of the vaccine, and 90% of the vaccine, with 15 min time intervals between doses. In the case of a positive SPT or IDT, each patient was proposed a titrated challenge with the same vaccine. Titration was performed according to the Nilson et al. desensitisation protocol: 4 or 5 doses were administered depending on the vaccine volume (0.005; 0.05; 0.1, 0.15 and 0.2 ml if needed), with 15 min time intervals between doses.^18^ All patients were then observed for at least 2 h and monitored by trained medical staff.

Skin tests (SPT and IDT) were also performed in a group of 16 healthy volunteers with no allergic symptoms post‐vaccination and negative drug allergy history. The data were analysed with the Statistica software. When comparing the two independent groups, the U Mann–Whitney test was used. Risk factors for vaccine skin sensitisations were assessed with a logistic regression model. The cut‐off point for the *p*‐value was 0.05. The study was approved by the appropriate Bioethics Commission (number NKBBN/166/2021).

## 3 RESULTS

Two hundred and eighy‐five adult patients were enrolled, 63 (22%) men and 222 (78%) women. The mean age was 52.7 (range 19–85). Through shared decision‐making, 221 (78%) patients initially decided to receive Comirnaty®, 9 – Spikevax®, 14 – Vaxzevria® and 41 – COVID‐19 Janssen ®.

**FIGURE 2:**
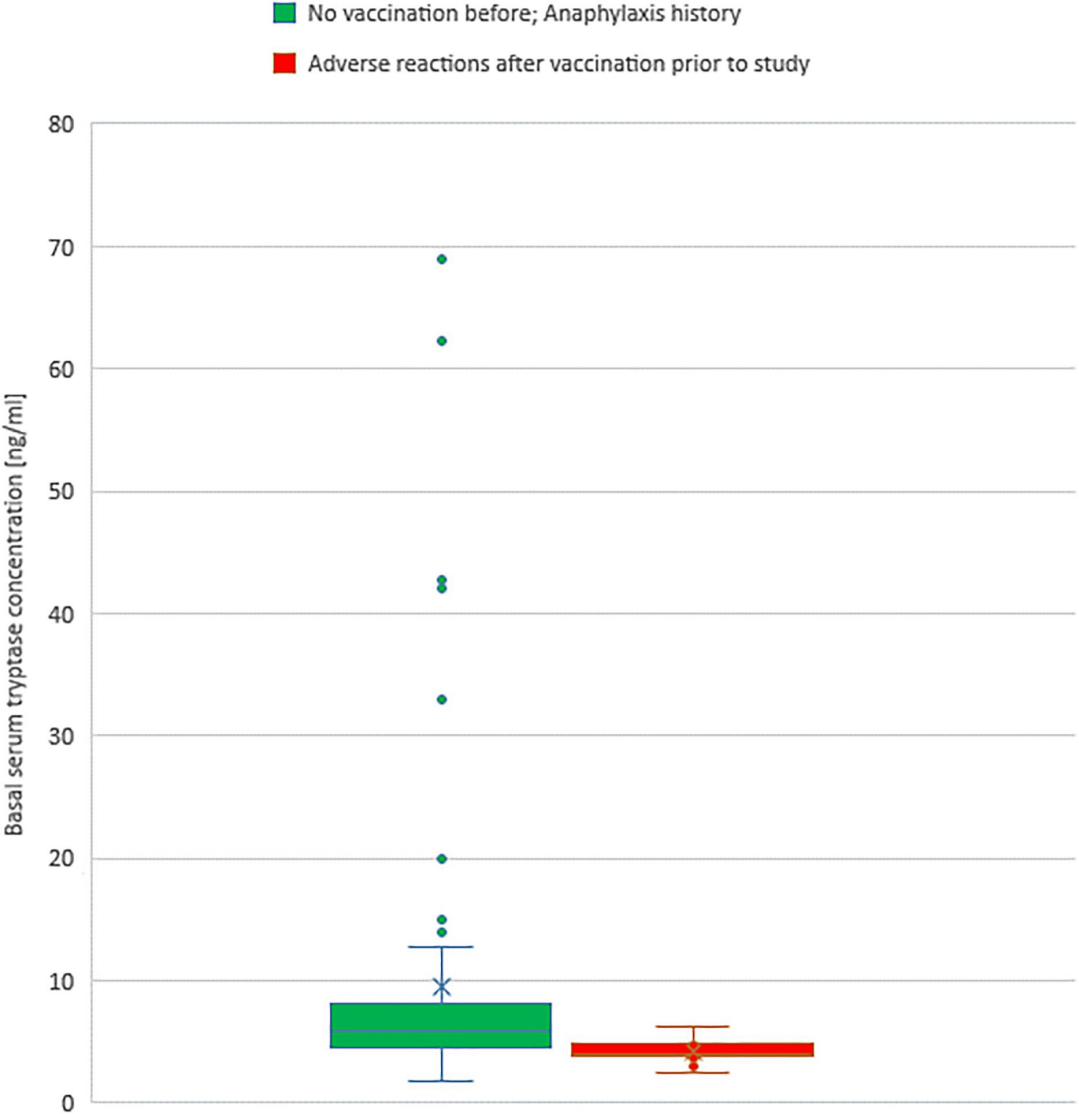
Comparison of basal serum tryptase concentration in patients’ reaction after the first dose of a vaccine and those enrolled based on anaphylaxis history. X mean, ― median; □ max; □ min; box quartile 25%–75%; □ outlier values. Five patients with tryptase above 20 ng/ml had mastocytosis diagnosed

Two hundred and five patients were enrolled due to severe allergy history and 80 experienced anaphylactic reactions after the first dose (of which, 54 received Comirnaty®, 4 – Spikevax® and Vaxzevria® – 22). The comorbidities in the two main patient groups are presented in Table 1. We found that patients with anaphylaxis risk (not yet vaccinated) more frequently had hypertension, diabetes, and obesity, and consequently higher risk of severe COVID‐19. In 79 patients, the serum tryptase concentration was measured. Patients with adverse reactions to the first dose of the vaccine before the study had a significantly lower basal serum tryptase concentration (*p* = 0.001) even when excluding the patients with mastocytosis from the statistics (*p* = 0.003; Table 1 and Figure 2).

**TABLE 1:** Comparison of the two groups of patients enrolled in the study

	Risk of anaphylaxis *N* = 205 (100%)	Reaction to first dose prior to study *N* = 80 (100%)	Total *N* = 285 (100%)	*p*‐Value
Women	160 (78%)	61 (76%)	221 (78%)	0.74
Men	45 (22%)	19 (24%)	64 (22%)	
Asthma	41 (20%)	10 (13%)	51 (18%)	0.099
Allergic rhinitis	36 (18%)	16 (20%)	52 (18%)	0.710
Hypertension	67 (33%)	10 (13%)	77 (27%)	<0.001
Coronary heart disease	18 (9%)	2 (3%)	20 (7%)	0.051
Hypothyroidism	32 (16%)	11 (14%)	43 (15%)	0.580
Diabetes	22 (11%)	3 (4%)	25 (9%)	0.049
Obesity	45 (22%)	7 (9%)	52 (18%)	0.005
Insect venom allergy	33 (16%)	9 (11%)	42 (15%)	0.239
Food allergy	17 (8%)	6 (8%)	23 (8%)	0.751
Mastocytosis or MCAS	6 (3%)	0 (0%)	6 (1%)	0.114
Mean serum tryptase *N* = 79	9.55 ng/ml	4.21 ng/ml	8.74 ng/ml	0.001 (0.003*)
Risk of severe COVID‐19 infection	122 (60%)	22 (28%)	144 (50%)	<0.001

*Note*: Risk of anaphylaxis was assessed according to the interview of anaphylaxis history to drugs or unknown allergen. The second group had an allergic reaction to the first dose of Comirnaty, Spikevax or Vaxzevria. MCAS, mast cell activation syndrome. *p*‐value was calculated with the Mann–Whitney *U* test. Red text shows significant values below 0.05. Serum tryptase was analysed in 79 randomly chosen patients. *Excluding patients with mastocytosis from analysis.

The choice of the vaccine was made according to Figure 1. However, in 28 cases despite the reaction after the first dose, the decision was made to continue with the same vaccine administration as the allergology specialist evaluated that the initial reaction did not include shock and the patient's decision was to continue with the same regimen.

In 278 patients, skin tests were negative. One of these patients withdrew consent for vaccination. In seven patients, skin tests were positive (Table 2) Food allergy showed a significant difference in these groups in the U Mann–Whitney test (*p* = 0.016).

**TABLE 2:** Summary of patients with positive skin tests.

Patient number	Gender	Age	Vaccine prior to study	Vaccine used for tests	Comorbidities	Known allergies	SPT result	IDT result	Titrated challenge	Complication
1	F	51	n/a	Spikevax	Asthma, allergic rhinitis	Drugs	‐	+	Yes	None
2	F	64	n/a	Vaxzevria	Asthma, allergic rhinitis	Hymenoptera, food, drugs	+	+	Yes	None
3	F	70	n/a	Janssen	None	Drugs	‐	+	Yes	None
4	F	39	Comirnaty	Comirnaty	Hypertension, hypothyroidism	Food, drugs	+	+	No consent	n/a
5	F	42	Comirnaty	Comirnaty	None	Drugs	‐	+	Yes	Mild reaction
6	F	70	n/a	Comirnaty	None	Drugs	+	+	yes	Mild reaction
7	M	77	n/a	Comirnaty	None	Drugs, Hymenoptera	‐	+	Yes	None

*Note*: Four patients had no vaccination previously and were enrolled based on anaphylaxis and allergy history. Eight patients developed non‐severe adverse symptoms to the first dose of a vaccine, so in a shared decision process, the same vaccine was selected. F, female; M, Male. Titrated challenge is performed according to desensitization protocol.^18^ +, positive test result; ‐, negative test result.

**FIGURE 3:**
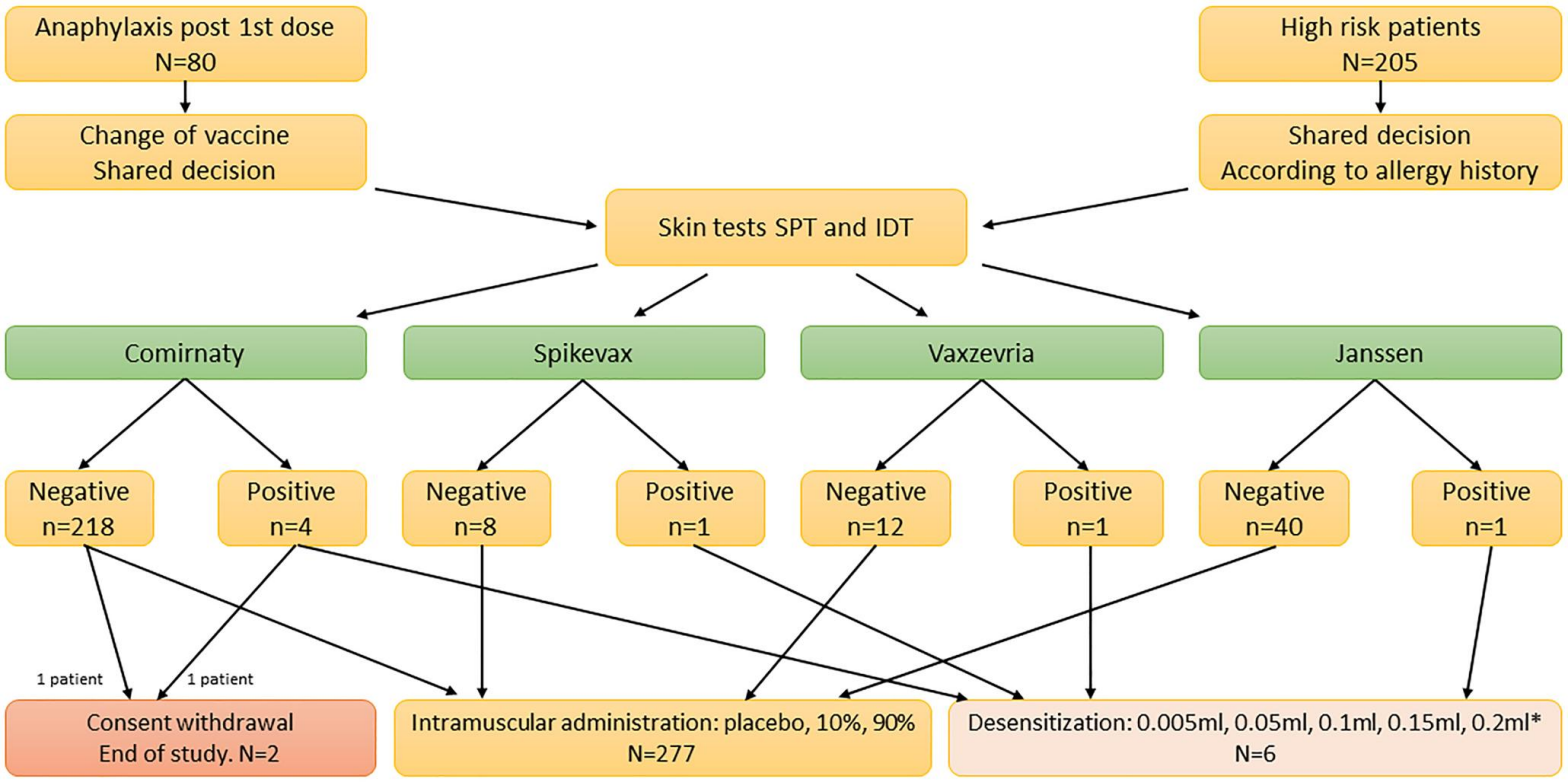
Results of allergy work‐up protocol. Patients entered the study in two groups: Anaphylaxis after the first dose, and high‐risk patients who had previously experienced severe allergic reactions to multiple groups of drugs or unknown allergens. All patients had skin tests performed. If the tests negative—intramuscular challenge with chosen vaccine was performed. If positive—desensitization was scheduled, although 2 patients withdrew consent at this point. IDT, intradermal tests; SPT, skin prick tests. *In Comirnaty, the final dose was not administered

Each patient with a positive test was offered to undergo titrated challenge with the anti‐COVID‐19 vaccine. One of them did not consent. In six cases, the procedure was performed. After completing the desensitisation, one of those patients received the same vaccine as initially, confirming that desensitisation functions in anti‐COVID‐19 vaccine allergy. In five cases, no adverse effects were observed, and in two cases mild adverse reaction (transient dyspnea with no impact on vital signs or physical examination) was noted that resolved quickly and did not require any treatment or hospitalisation (Table 2 and Figure 3).

Eventually, 283 people were vaccinated. In this group, 144 (50%) patients had risk factors for comorbidities of severe COVID‐19, however, the management according to protocol enabled safe vaccination of all of those who had given informed consent. The results of the allergy work‐up protocol and the outcome of the study for all patients is presented in Figure 3.

In the control group, SPT and IDT were performed with Comirnaty and Janssen. All results were negative and no skin irritation was observed.

## 4 DISCUSSION

The main finding in our study was to establish a safe anti‐COVID‐19 vaccination protocol in the high‐risk population of patients with prior anaphylactic reactions and with adverse reactions after the first dose of anti‐COVID‐19 vaccines. This is in line with the clinical trials of Comirnaty®, Spikevax®, Vaxzevria® and COVID‐19 Janssen®.^2^, ^3^, ^4^, ^5^ According to the available data from the clinical trials, hypersensitivity or allergy to these vaccines is extremely rare—for Comirnaty® was assessed as 0.6%, for Spikevax ®—1.5%, for Vaxzevria® and COVID‐19 Janssen®—<0.1%.^2^, ^3^, ^4^, ^5^ The numbers differ mostly due to the different reporting methodologies between the trials and in most cases were similar to the placebo. Although these rates are small compared to most allergenic drug groups such as penicillins, they are still notably higher than other vaccines introduced much earlier such as anti‐Influenza or anti‐Hepatitis B, as shown by Nilsson et al.^18^ We are aware that this might be due to the different methodologies in these studies.

Real‐life survey studies show that allergic reactions are reported four times more frequently by people with allergy histories (0.2%).^23^ However, according to our experience, many patients consider common side effects to be an allergic reaction,^24^, ^25^ and it is difficult to distinguish those especially if no documentation is available.

Patients who have a history of anaphylactic reactions frequently present increased anxiety on receiving any drug.^26^ Those who experienced an adverse reaction to the first dose of an anti‐COVID‐19 vaccine are particularly afraid of the second dose of vaccination. Often, those patients question physicians who explain that there is no definite contraindication to the vaccines. The result of the study might suggest that there is no point in actually performing skin tests (SPT, IDT) with anti‐COVID‐19 vaccines if nearly all of them are negative both in the study and control groups. Still, as the experience of allergologists indicates, in the shared decision‐making process, any help in comforting the patient is significant. As expected, allergy work‐up lowers patients' stress.^27^ Anxious patients are usually more willing to receive the vaccine after negative tests associated with close monitoring by an allergologist. Therefore, even though skin testing does not always significantly affect the physician's decision, it does help in the vaccination process as part of the allergy work‐up.

Similarly to other studies such as Rasmussen et al., our results show that even patients with severe disturbing reactions after the first dose of a vaccine can be safely evaluated, and nearly all of them can receive a second dose.^28^ However, our study went even further, offering titrated challenge to people with positive skin tests with significant patient cooperation (six out of seven) combining reasonable safety and vaccination success. We agree that application of desensitisation protocol without previous reactions to the tested drug is controversial. However, these patients with positive skin tests would not agree to receive a vaccine in a standard way. Thus, offering a ‘special’ desensitising protocol increases the number of those eventually vaccinated, which was our primary goal. Finally, there was a single patient with confirmed Comirnaty allergy (Table 2, subject 5) who received a successful desensitisation.

The question of testing with either the entire vaccine or it's excipient (PEG, pokysorbat 80) remains open. Barbaud et al.‘s recommendations suggest skin tests with excipients in patients with a high risk of vaccine allergy.^12^ The group of patients who developed anaphylaxis after the first dose should also receive a SPT with an anti‐COVID‐19 vaccine. Our study shows that IDT with all four anti‐COVID‐19 vaccines is also a viable option. Some studies suggest that standard vaccination might induce a positive delayed reaction to skin testing.^29^ Our study showed that there were no significant blistering or edema on the test area for the previously vaccinated patients or healthy volunteers. These delayed reactions obviously would not influence the vaccination decision, as the results of the SPT and IDT were determined after 15 min. Furthermore, delayed reactions in those tests is of doubtful clinical significance.

It is also highly likely that the broad media discussion plays a role in the peoples' choices of the type of vaccine. It was significant that 78% with no previous post‐vaccine reaction, who could freely choose a vaccine, decided to receive Comirnaty®. Many of them were afraid of Vaxzevria's® adverse events, namely the rare thrombosis that was discussed in the European media recently.^30^ Even facing proper explanations from an allergologist that the benefits far exceed the risks, this prejudice persisted.^31^

No gender selection was applied on enrolment, however women still significantly prevailed in the studied group (78%). This is in line with other studies emphasising a higher prevalence of drug allergy in women. Macy and Ho analysed 2,375,424 patient records. In this large group, 20% of patients reported drug allergies, of which 68% were women.^32^, ^33^ This discrepancy is generally observed after puberty. Though its cause remains unknown, the possible reason might include the higher drug use by female individuals, which results in greater potential allergen exposure. Additional causes are also considered: epigenetics of the X chromosome and the impact of sex hormones.^33^

Surprisingly, our result suggested that patients with hypersensitivity reactions to anti‐COVID‐19 vaccines have lower tryptase levels (Table 1). Of course, the result should be treated with caution due to the small number of patients tested. Further, this is difficult to compare these results with other studies. European Competence Network on Mastocytosis (ECNM), in a preliminary report, shows that the prevalence of general hypersensitivities in patients with mast cell disorders decreases in high concentrations of serum tryptase.^34^ However, it must be noted that ECNM did not take into account vaccines. These are extremely difficult to evaluate in such a registry due to the low vaccine allergy rate and rare administration. Nevertheless, the possibility of a relationship between low tryptase level and increased reaction to an anti‐COVID‐19 vaccine should be considered a risk factor in future trials.

Although our study shows a relationship between skin sensitisation and food allergy, the group with positive tests is still relatively small. Furthermore, a logistic regression model did not reveal any significant correlation that would predict positive skin tests. Larger groups are required to investigate this.

## 5 CONCLUSIONS

SPT and IDT with Comirnaty®, Spikevax®, Vaxzevria® and COVID‐19 Janssen® are a useful tool in the COVID‐19 vaccination protocol. This protocol enables safe immunisation of high‐allergy‐risk patients and relatively safe desensitization in cases of positive tests. High basal serum tryptase probably should not be considered to be a risk factor of anti‐COVID‐19 vaccine allergy as allergic reactions occur more frequently in low concentrations.

## AUTHOR CONTRIBUTIONS

**Jan Romantowski:** Conceptualization; Equal, Data curation; Equal, Formal analysis; Equal, Investigation; Equal, Methodology; Equal, Project administration; Equal, Resources; Equal, Software; Equal, Writing—original draft; Equal, Writing—review and editing; Equal. **Jerzy Kruszewski:** Conceptualization; Equal, Data curation; Equal, Investigation; Equal, Methodology; Equal, Resources; Equal, Supervision; Equal, Writing—review and editing; Equal. **Oskar Solarski:** Data curation; Equal, Funding acquisition; Equal, Investigation; Equal, Methodology; Equal, Resources; Equal, Software; Equal, Writing—review and editing; Equal. **Andrzej Bant:** Data curation; Equal, Investigation; Equal, Resources; Equal, Software; Equal, Writing—review and editing; Equal. **Andrzej Chciałowski:** Conceptualization; Equal, Data curation; Equal, Investigation; Equal, Project administration; Equal, Resources; Equal, Software; Equal, Supervision; Equal, Writing—review and editing; Equal. **Ilona Pietrzyk:** Data curation; Equal, Investigation; Equal, Resources; Equal, Software; Equal, Writing—review and editing; Equal. **Patrycja Sańpruch:** Data curation; Equal, Investigation; Equal, Resources; Equal, Software; Equal, Writing—review and editing; Equal. **Aleksandra Górska:** Data curation; Equal, Investigation; Equal, Resources; Equal, Software; Equal, Writing—review and editing; Equal. **Marta Chełmińska:** Data curation; Equal, Investigation; Equal, Methodology; Equal, Resources; Equal, Software; Equal, Writing—review and editing; Equal. **Agata Knurowska:** Data curation; Equal, Investigation; Equal, Methodology; Equal, Resources; Equal, Software; Equal, Writing—original draft; Equal, Writing—review and editing; Equal. **Marika Gawinowska:** Data curation; Equal, Investigation; Equal, Methodology; Equal, Resources; Equal, Software; Equal, Writing—review and editing; Equal. **Ewa Jassem:** Conceptualization; Equal, Data curation; Equal, Formal analysis; Equal, Investigation; Equal, Methodology; Equal, Project administration; Equal, Resources; Equal, Supervision; Equal, Validation; Equal, Visualization; Equal, Writing—review and editing; Equal. **Marek Niedoszytko:** Conceptualization; Equal, Data curation; Equal, Formal analysis; Equal, Funding acquisition; Equal, Methodology; Equal, Project administration; Equal, Resources; Equal, Supervision; Equal, Validation; Equal, Writing—review and editing; Equal.

## CONFLICT OF INTEREST

The authors declare no conflict of interests.
